# An assessment of forward and inverse GIA solutions for Antarctica

**DOI:** 10.1002/2016JB013154

**Published:** 2016-09-29

**Authors:** Alba Martín‐Español, Matt A. King, Andrew Zammit‐Mangion, Stuart B. Andrews, Philip Moore, Jonathan L. Bamber

**Affiliations:** ^1^ School of Geographical SciencesUniversity of BristolBristolUK; ^2^ School of Land and FoodUniversity of TasmaniaHobartTasmaniaAustralia; ^3^ Centre for Environmental Informatics, National Institute for Applied Statistics Research Australia (NIASRA)University of WollongongWollongongNew South WalesAustralia; ^4^ School of Civil Engineering and GeosciencesNewcastle UniversityNewcastle upon TyneUK

**Keywords:** GIA

## Abstract

In this work we assess the most recent estimates of glacial isostatic adjustment (GIA) for Antarctica, including those from both forward and inverse methods. The assessment is based on a comparison of the estimated uplift rates with a set of elastic‐corrected GPS vertical velocities. These have been observed from an extensive GPS network and computed using data over the period 2009–2014. We find systematic underestimations of the observed uplift rates in both inverse and forward methods over specific regions of Antarctica characterized by low mantle viscosities and thin lithosphere, such as the northern Antarctic Peninsula and the Amundsen Sea Embayment, where its recent ice discharge history is likely to be playing a role in current GIA. Uplift estimates for regions where many GIA models have traditionally placed their uplift maxima, such as the margins of Filchner‐Ronne and Ross ice shelves, are found to be overestimated. GIA estimates show large variability over the interior of East Antarctica which results in increased uncertainties on the ice‐sheet mass balance derived from gravimetry methods.

## Key Points

- Analysis and assessment of forward and inverse GIA solutions for Antarctica
- Identification of regions where GIA solutions systematically underpredict/overpredict the uplift rates
- Large variability over the interior of East Antarctica result in increased uncertainties on the ice‐sheet mass balance

## 1 Introduction

Estimating the mass balance of the ice sheets and their current contribution to sea‐level rise is a challenging research task in Earth Sciences. The Gravity Recovery and Climate Experiment (GRACE) observes temporal gravity variations from which surface mass changes over a given region can be deduced. However, GRACE is sensitive to vertically integrated mass changes. Therefore, it is not possible to distinguish between mass changes caused by present‐day ice‐mass variations and other superimposed mass signals like those associated with glacial isostatic adjustment (GIA), the ongoing response of the solid Earth to past changes in ice‐ocean surface loading. In order to estimate the mass balance of ice sheets from gravimetry methods, the contribution from GIA must be accounted for. GIA is especially important over regions where ice sheets have thinned since the Last Glacial Maximum (LGM) about 20,000 years ago. GIA results in what is commonly considered to be a secular change (over timescales of centuries) in surface deformation and gravity field. It is normally accounted for using forward models which combine deglaciation history models and viscoelastic Earth structure models [e.g., *Sasgen et al.*, 2007; *Velicogna and Wahr*, 2006]. Alternatively, GIA uplift rates can be inferred from an inversion of satellite altimetry and gravimetry measurements with assumptions related to surface density changes [e.g., *Riva et al.*, 2009; *Gunter et al.*, 2014]. We will refer to these as inverse methods. Hence, the GRACE‐deduced mass change associated with climate and hydrology changes is strongly dependent on the GIA model used.

Forward GIA models inherit uncertainties from uncertainties in the viscoelastic rheology of the solid Earth and uncertainties in defining the spatial and temporal evolution of the ice load. The time‐scale of the load that needs to be defined is a function of mantle viscosity and is typically thought to be dominated by LGM loading changes [e.g., *Peltier*, 2004]. However, such changes are less relevant in regions of low mantle viscosity and more recent changes need to be considered [e.g., *Nield et al.*, 2014]. GIA modeling studies have demonstrated the sensitivity of GIA predictions to inferred 3‐D variations in solid Earth rheology [*van der Wal et al.*, 2015; *Geruo et al.*, 2013; *Kaufmann et al.*, 2005]. Conventional forward GIA models for Antarctica are based on sparse ice extent data (in space and time), and, together with the uncertain solid Earth rheology, this results in large uncertainties in predicted present‐day deformation of gravity field changes. However, these models are not commonly provided with complete uncertainty estimates [see *Whitehouse et al.*, 2012b, for a partial exception].

Inverse methods, on the other hand, reduce the dependence on numerical models, aiming to identify the GIA signal in GRACE and altimetry observations, in particular, and possibly also combined with GPS measurements of vertical surface deformation. They have provided new estimates of GIA with quantifiable uncertainties and have led to refinements of ice‐load histories to be used in forward models [*Milne et al.*, 2004; *Steffen and Kaufmann*, 2005]. However, they are highly dependent on surface density change assumptions, are restricted by the low resolution of GRACE data, and are limited by altimetry data coverage in areas of high relief.

In this study, we present an assessment of the most recent forward and inverse GIA solutions available for Antarctica, both on continental and regional scales, based on a comparison with a set of elastic‐corrected observed vertical velocities from GPS data. We aim to determine similarities and differences from the available solutions, identifying biases and errors relative to specific GIA models.

### 1.1 Forward Models of Glacial Isostatic Adjustment

GIA solutions based on forward modeling require two main inputs: a deglaciation history model and a model for the viscoelastic solid Earth structure and rheology. Different combinations of these two components together with different parameter assumptions in each application have led to a variety of GIA solutions which are currently available for Antarctica. Deglaciation models describe the temporal and spatial distribution of ice on the Earth's surface since LGM, with deglaciation assumed to terminate in the range of 2–6 ka before present, depending on the model [*Argus et al.*, 2014; *Whitehouse et al.*, 2012b; *Ivins et al.*, 2013]. For Antarctica, they show a wide variability in the amplitude and distribution of mass losses since the LGM, due to the limited number of ice extent constraints available in Antarctica [*Bentley et al.*, 2014]. Here we focus on the three main deglaciation models used nowadays: (i) ICE‐6G_C [*Peltier et al.*, 2015; *Argus et al.*, 2014], (ii) W12 [*Whitehouse et al.*, 2012b], and (iii) IJ05_R2 [*Ivins et al.*, 2013]. While ICE‐6G is a global ice‐history model, W12 and IJ05‐R2 are regional ice histories for Antarctica, supplemented by ICE‐5G [*Peltier*, 2004] outside of Antarctica. The total meltwater contribution from Antarctica since the LGM is larger in the ICE‐6G_C model, with 13.6 m of eustatic sea level (ESL), than in the IJ05_R2 and W12 models where the ice loss since the LGM amounts to 7.5 m and 8 m ESL, respectively.

Earth rheology and structure govern the solid Earth response to surface load changes over time. Most Earth models only consider variations of viscosity with depth and adopt a linear Maxwell rheology. However, recently researchers have developed models based on finite elements for treating three‐dimensional variations of the Earth structure, considering, in particular, lateral variations in effective viscosity and in some cases power law rheologies [*Geruo et al.*, 2013; *van der Wal et al.*, 2015].

The different models, detailed below, are summarized in Table 1.

**Table 1:** Summary of Main Parameters of the Forward GIA Models Used in This Study^a^

Model Units	*η*_*UM*_×10^21^ Pa s	*η*_*LM*_×10^21^ Pa s	LT (km)	ESL (m)
ICE‐6G_C (VM5a)	0.5	10	100	13.6
W12	1	10	120	8
IJ05_R2 (strong)	0.2	4	115	7.5
IJ05_R2 (weak)	0.2	1.5	65	7.5
A13	b	b	b	17.5
AGE1b	0.4–0.8	2–20	c	–

a *η*_*UM*_ and *η*_*LM*_ are the upper and lower mantle viscosities; LT is the lithosphere thickness.

b The 3‐D viscosity profile is determined from a global seismic tomography model (*η*_*UM*_ values ranging between 0.5 and 1.5 × 10^21^ Pa s) along with a model of elastic lithosphere thickness (values ranging between 130 km in the interior of East Antarctica to 30 km over the northernmost tip of the Antarctic Peninsula and part of the Ross Ice Shelf; see Figure 9 in *Geruo et al.* [2013]).

c AGE1b is based on a spatially varying lithospheric thickness (60 km for the Antarctic Peninsula, 150 km for East Antarctica, and 100 km for West Antarctica).

#### 1.1.1 W12 [*Whitehouse et al.*, 2012b]

The *Whitehouse et al.* [2012b] GIA solution is driven by the W12 deglaciation history [*Whitehouse et al.*, 2012a], which was constructed using an ice‐sheet model and tuned to attempt to fit 62 ice thickness observations, derived from exposure age dating information, many of which were not available in previous models [e.g., ICE‐5G *Peltier*, 2004]. The total ESL contribution since the LGM amounts to 8 m and occurs slow and monotonically over 20 ka, with no melt after 2 ka BP. The W12 Earth model, described in *Whitehouse et al.* [2012b] was tuned using observations of relative sea‐level (RSL) change and marine limit observations around Antarctica to obtain a best fit combination of viscosity of the mantle and lithospheric thickness, finding a strong dependency to the upper‐mantle viscosity with values varying in the range of 0.5–2 × 10^21^ Pa s. The best fitting model values are shown in Table 1. To deal with the overall larger GPS rates in the Antarctic Peninsula with respect to the observations and unconsidered accumulation increases in this region over recent centuries [*Nield et al.*, 2012], *Whitehouse et al.* [2012b] produce a variant called W12a which includes 150 m of ice gain from 1000 to 500 years ago and a further 150 m of ice gain from 500 to 100 years ago. These modifications cause a decrease in the mean bias for the full set of sites from 1.8 to 1.2 mm yr^−1^. Subsequently, this modification was considered an overcorrection and is not widely used [*Wolstencroft et al.*, 2015].

#### 1.1.2 IJ05_R2 [*Ivins et al.*, 2013]

The ice history model IJ05_R2 incorporates ice extent constraints (from ice cores, rock exposure dating, and palaeogrounding line positions) similar to those used to produce W12, leading to a reduction of the total meltwater volume expulsion since the LGM compared to the earlier models IJ05 [*Ivins and James*, 2005] and ICE‐5G [*Peltier*, 2004]. The total volume of ice at the LGM was 7.5 m ESL. The majority of ice loss occurs later than in other models such as W12 and ICE‐6G_C, and deglaciation ended at 1 ka BP. They consider two different Earth models (defined in Table 1), one of which has a strong lithosphere, consistent with East Antarctica, and a second with a thinner lithosphere and weaker lower mantle viscosity yielding faster uplifts. We use the stronger of the two Earth models in this assessment.

#### 1.1.3 ICE‐6G_C (VM5a) [*Peltier et al.*, 2015; *Argus et al.*, 2014]

ICE‐6G_C ice history is a globally consistent ice history model developed by fitting ice extent to geodetic and RSL data. In Antarctica, the same 62 ice extent sites as used in W12 were adopted with GPS uplift rates also used to define the ice history. Some of the GPS sites used are the same as those we use in this study. The total post‐LGM sea level contribution is 13.6 m ESL, with deglaciation terminating at 4 ka BP. ICE‐6G_C (VM5a) has been modified from its predecessor ICE‐5G (VM2) [*Peltier*, 2004] to, among other things, improve the fit to GPS uplift rates and other geological observations in Antarctica. These modifications result in a reduction of 4 m in the total amount of ice loss since the LGM compared to ICE‐5G (which accounted for 17.5 m ESL). Unlike the other forward models considered here, the ICE‐6G ice history was developed in conjunction with an Earth model (VM5a), with parameters given in Table 1. Predictions of the current GIA uplift from the ICE‐6G_C (VM5a) model were downloaded from http://www.atmosp.physics.utoronto.ca/∼peltier/data.php (last accessed 3 August 2016). This corresponds to the latest set of revised spherical harmonic coefficients made available on the group's website, after a review of those originally published. Following the initial submission of this manuscript, *Purcell et al.* [2016] pointed out an anomalously large uplift signal in the ICE‐6G_C model over regions where ice has been grounded below sea level at or since the LGM. They produced a new data set (ICE6G_ANU) of present‐day GIA signal based on the same ice history and Earth rheology which is shown to better represent the change in surface load in those areas. However, this has not been used in this study.

#### 1.1.4 A13 [*Geruo et al.*, 2013]

*Geruo et al.* [2013] present an advance to the GIA modeling by developing a finite element method to solve numerically for the 3‐D viscous response of a compressible Earth to surface loading. Therefore, they use a viscoelastic profile that varies laterally, with variations in elastic lithosphere thickness and mantle viscosities, unlike the other forward models considered here (such as in W12, IJ05‐R2, or ICE‐6G). The Earth model was forced with the ICE‐5G deglaciation model. This deglaciation model assumes large ice losses since the LGM (17.5 m ESL). The A13 GIA model output was downloaded from ftp://podaac-ftp.jpl.nasa.gov/allData/tellus/L3/pgr/ (last accessed 9 March 2016).

#### 1.1.5 AGE1b [*Sasgen et al.*, 2013]

AGE‐1b is an hybrid approach that involves the use of a set of three different loading histories and four viscosity distributions, two different elastic corrections (based on the input‐output method and ICESat) for the GPS uplift rates, and two GRACE solutions (for which they estimate linear trends for January 2003 to September 2012) combined through a two‐step statistical procedure. First, they perform a first‐order global inversion by fitting, in a least squares approach, the ensemble of GIA forward models (derived from forcing the four Earth models with the three loading histories) to the peak signal in the GRACE trends. In a second step, they fit GPS rates to the forward model ensembles for different sectors in Antarctica. Finally, they calculate the arithmetic mean of the ensemble to estimate their best GIA estimate for Antarctica. Out of the three ice‐loading histories used in AGE1b [IJ05, *Ivins and James*, 2005 H02, *Huybrechts*, 2002 and ICE‐5G(VM2) *Peltier*, 2004], two of these (IJ05 and ICE‐5G) have been recently superseded in IJ05‐R2 and ICE‐6G. Range of values for the Earth model are given in Table 1.

### 1.2 Inverse Models of Glacial Isostatic Adjustment

#### 1.2.1 R09 [*Riva et al.*, 2009]

The work described in *Riva et al.* [2009] represents the first attempt to separate the deformation caused by surface processes (ice and firn) from that of the solid Earth (GIA) using a combination of ICESat (March 2003 to March 2008) and GRACE data sets. Their methodology is based on the principle of mass conservation and relies on the difference in density between snow/ice and the solid Earth [*Wahr et al.*, 2000]. To estimate mass changes from altimetry, they use a surface snow density map (ranging 320–450 kg m^−3^ [*Kaspers et al.*, 2004]) and the density of ice (917 kg m^−3^) in regions with balance velocities larger than 25 m yr^−1^. Firn densification processes are not accounted for in this study. To account for the elastic response of the solid Earth to present‐day changes in surface load, they upscale the height changes obtained using ICESat by 1.5%.

#### 1.2.2 G14 [*Gunter et al.*, 2014]

*Gunter et al.* [2014] adapt the methodology described in *Riva et al.* [2009] to account for regional variations in accumulation rates. They use values provided from the regional climate model (RACMO2) and firn densification model [*Ligtenberg et al.*, 2011]. This allows to account for changes in height due to firn densification processes, which was a limiting factor in the earlier study. They computed and assessed a total of 10 different solutions based on different GRACE solutions. Then, they uniformly applied a “low‐precipitation zone” bias correction (“LPZ bias”) to the GIA solution to match precipitation rates in East Antarctica. The solution we have used to compare against others is that based on the CSR RL05 DDK5 fields. This is a regularized solution based on Release‐05 GRACE Level‐2 data product developed by the Austin Center for Space (CSR) which is done through a Wiener‐type filter [see *Gunter et al.*, 2014].

#### 1.2.3 RATES [*Martín‐Español et al.*, 2016]

*Martín‐Español et al.* [2016] presented a GIA solution from an inversion of altimetry, gravimetry, and GPS data, as part of the Resolving Antarctic ice mass TrEndS (RATES) project. They employed a spatiotemporal Bayesian hierarchical model to simultaneously estimate the different latent processes occurring in the ice sheet (surface mass balance, firn compaction, ice dynamics, and a time‐invariant GIA). The approach relies on the different spectral spatiotemporal characteristics of the latent processes involved in the overall height change, which were extracted from numerical model outputs. In particular, they used IJ05_R2 for an initial estimate of the spatial wavelength of GIA over East Antarctica (1700 km) reduced over West Antarctica (500 km).

## 2 GPS Data and Elastic Correction

In this section we outline the data used for carrying out the comparison and the corrections we apply for it to be representative of GIA.

### 2.1 GPS Analysis Methods

**Figure 1:**
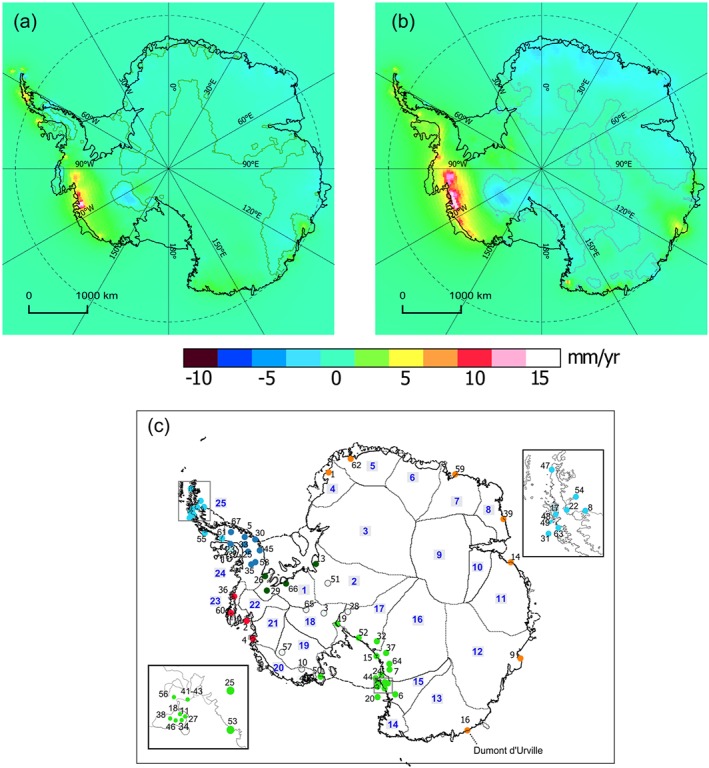
(a and b) The mean elastic response estimated from the Regional ElAstic Rebound Calculator using the loadings from *Martín‐Español et al.* [2016] for the periods 2003–2009 and 2010–2013, respectively. The zero contour line is shown. (c) The GPS stations used in this analysis. Each station is identified with a number and a color. Station numbers are indexed in Table S1. Colors are used to distinguish the different subregions which are used in Table 2. Stations marked in light blue represent those within the North Antarctic Peninsula, dark blue those within the South Antarctic Peninsula, red those within the Amundsen Sea Embayment, dark green those within the Filchner‐Ronne Ice Shelf, light green those within the Ross Ice Shelf, and orange those along coastal East Antarctica. Basin definitions are those of *Sasgen et al.* [2013], and their number is shown in blue.

GPS data were obtained from public and private archives for the sites shown in Figure 1. We only consider data from 2009 to 2014 inclusive. The exact observation period used and other related information for each GPS site can be found in the supporting information. We processed the data in NASA Jet Propulsion Laboratory (JPL) GIPSY v6.3 software using a precise point positioning approach [*Zumberge et al.*, 1997] using JPL clocks, orbits, and Earth orientation parameters. Our analysis was quite standard, estimating one site coordinate per day, and tropospheric zenith delay, horizontal gradients, and receiver clock terms every measurement epoch. We mapped the estimated zenith tropospheric delays to the elevation of each satellite using the Vienna Mapping Functions 1 [*Boehm et al.*, 2006] with a priori hydrostatic zenith delays derived from European Centre for Medium‐Range Weather Forecasts model fields [*Tregoning and Herring*, 2006]. We applied absolute antenna phase center corrections [igs08_1758.atx; *Schmid et al.*, 2015] and corrected for second order ionospheric terms using JPL ionosphere grids and a shell height of 600 km [*Petrie et al.*, 2011]. Solid Earth tides were modeled using IERS2010 standards, and ocean tide loading displacements were modeled using the TPXO7.2 tide model and SPOTL software in the center of mass of the whole Earth system frame [*Agnew*, 1997; *Egbert et al.*, 2009; *Fu et al.*, 2012; *Petit and Luzum*, 2010]. In postprocessing we removed erroneous data, including those apparently affected by snow on the radome [*Argus et al.*, 2014; *Wolstencroft et al.*, 2015], subtracted the effects of atmospheric pressure loading displacements based on the Modern Era Retrospective‐analysis for Research and Applications (MERRA) atmospheric pressure field computed at http://massloading.net. We also removed the annual effects of elastic deformation due to ice load changes, as described below, prior to trend estimation. GPS vertical velocities were estimated using the CATS software [*Williams*, 2008], estimating the trend alongside semiannual and annual periodic terms and offsets where required (due to changes in receiver make or antenna), while considering a white‐plus‐flicker noise model [*Williams*, 2003] to account for temporal correlation in the time series. We note that the effects of postseismic deformation are not well known [*King and Santamaría‐Gómez*, 2016], but in the vertical component they appear limited to close to the location of the 1998 magnitude 8.2 earthquake north of Dumont d'Urville. For that location we do not use the GPS velocity and instead adopt the pre‐1998 DORIS velocity computed by *King and Santamaría‐Gómez* [2016].

### 2.2 Elastic Deformation

Present‐day melting of ice mass loads causes instantaneous, as well as long‐term responses, in the solid Earth system. While the elastic response is immediate and concentrated relatively close to the load changes, the viscoelastic response of the mantle takes place on longer timescales (from decadal to millenial) and is spatially smooth over longer (often hundreds of kilometer) spatial scales. Elastic uplift caused by present‐day ice mass loss can be an order of magnitude larger than the underlying viscoelastic signal and can lie in the range of several centimeters per year [*Thomas et al.*, 2011; *Nield et al.*, 2014]. If GPS velocities are to be used to test models of GIA, as done here, it is highly important to consider and correct the GPS measurements for elastic deformation before using them to test or constrain GIA models. Elastic deformation computations require a high‐resolution model of surface mass variation that overlap in time with the GPS data period in order to obtain accurate GPS velocity corrections. Here we model the annual elastic vertical deformations caused by ice mass changes on Antarctica over the 2003–2013 period (but only elastic estimates from 2009 to 2013 are used to correct the GPS validation set; see section 3.2). The ice mass trends used to estimate the elastic correction are derived from a rigorous statistical combination of satellite gravimetry (GRACE), altimetry (ICESat, Envisat, and Cryosat‐2) and GPS within a Bayesian framework following *Zammit‐Mangion et al.* [2015a, 2015b]; *Martín‐Español et al.* [2016]. A different mass balance estimate for Antarctica is used to obtain an alternative elastic correction later in section 4.4.2 and test the sensitivity of our results. The effects of these present‐day loading changes on the vertical motion of the Earth's crust were estimated using the Regional ElAstic Rebound Calculator (REAR) [*Melini et al.*, 2015]. In order to compute the surface rates of displacement associated to a unit rate of mass variation (Green functions), REAR uses a set of load deformation coefficients derived from the seismological Earth model STW105 [*Kustowski et al.*, 2008] up to harmonic degree 30,000 (<1 km spatial resolution). We use a disc‐shaped load function with a constant radius of approximately 10 km. Vertical displacements were calculated following the methods described in *Melini et al.* [2015] and used to correct the GPS trends.

The elastic correction covaries with mass balance and hence has strong interannual variations. Figure 1 illustrates the mean elastic trends over the time periods 2003–2009 and 2010–2013. The Amundsen Sea Embayment (ASE) presents the largest ice losses of the whole Antarctic continent, and as a consequence, it experiences the strongest elastic response in our model with maximum uplift rates of 16 and 19 mm yr^−1^ over 2003–2009 and 2010–2013, respectively, due to increased mass losses in the region between the two periods [see *Martín‐Español et al.*, 2016, Table 2]. The elastic response over the South Antarctic Peninsula (SAP) presents a shift from subsidence to uplift rates between the two periods, as the destabilization of glaciers draining to the Bellingshausen Sea takes place [*Wouters et al.*, 2015]. We note that our modeled elastic uplift in the North Antarctic Peninsula (NAP) is smaller than that of *Nield et al.* [2014], likely due to their use of very high resolution mass change data sets. The Kamb Ice Stream is the only region where we observe systematic mean subsidence rates primarily as a consequence of the persistent dynamic thickening rates found over this glacier [*Retzlaff and Bentley*, 1993]. In East Antarctica, the elastic signal modeled for the period 2003–2009 is rather small and specific to glaciers near the coastline of Wilkes Land. However, GRACE data suggest that for the period 2010–2013, the coast of Queen Maud Land, East Antarctica, accumulated snow at 150 Gt yr^−1^ [*Argus et al.*, 2014; *Boening et al.*, 2012]. This caused the subsidence of about 2 mm yr^−1^ according to the elastic model output from REAR with modeled rates at nearby GPS sites *VESL* (−2.4 mm yr^−1^), *ABOA* (−0.7 mm yr^−1^), *SYOG* (−0.5 mm yr^−1^).

## 3 Results

### 3.1 Solutions for Glacial Isostatic Adjustment in Antarctica

**Figure 2:**
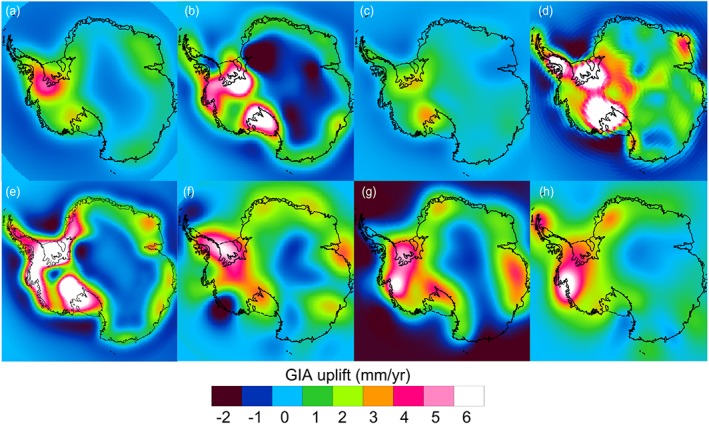
GIA uplift rates estimated from different solutions for Antarctica in mm yr^−1^: (a) IJ05_R2, (b) W12, (c) AGE‐1b, (d) A13, (e) ICE‐6G_C (VM5a), (f) R09, (g) G14, and (h) RATES.

**Figure 3:**
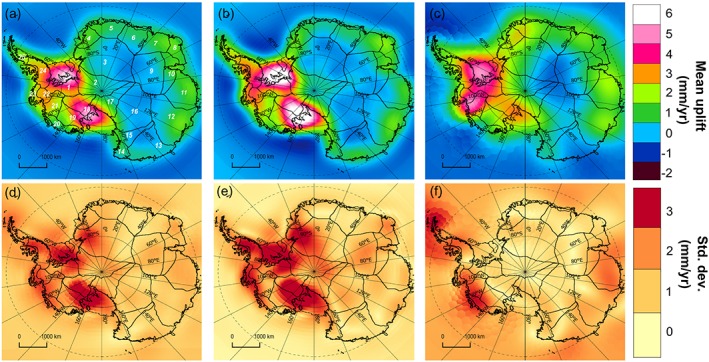
(a) Mean and (d) standard deviation of the uplift rates in mm yr^−1^ of the eight GIA solutions included in this study and (b, e) separate fields for the forward and (c, f) inverse models.

The various forward and inverse models of present‐day GIA uplift rates are shown in Figure 2. GIA uplift rates show a large variability across the different solutions, even when considering inverse approaches and forward models separately. There is a clearly distinctive large‐scale pattern common to all solutions which becomes evident when computing the mean from all the GIA estimates (Figure 3a). High uplift rates are characteristic in West Antarctica, while uplift rates have a smaller magnitude in coastal East Antarctica and the interior presents regions of subsidence of variable size across solutions. The large‐scale pattern agrees well with inferences of low mantle viscosities in West Antarctica and higher values in East Antarctica [*An et al.*, 2015; *van der Wal et al.*, 2015]. The latter is expected to differ by at least 2 orders of magnitude between East and West Antarctica, being larger over East Antarctica [*van der Wal et al.*, 2015]. We note that the choice of spatially varying correlation lengths for the RATES GIA solution was also inferred from this information, and hence, it is not free of assumptions. Additionally, we compute the mean and standard deviation of inverse and forward models separately (Figures 3b and 3c, respectively). Forward models predict their uplift maxima over the Filchner‐Ronne (FRIS) and Ross Ice Shelves while the inverse methods' largest uplifts are found over the FRIS and the ASE. Over East Antarctica, forward models estimate a larger area of subsidence than inverse models. The spatial variability between all solutions is in many cases larger than the GIA signal itself, especially in areas of the interior of East Antarctica where mean GIA rates are small. The largest variability is found over the FRIS, the western margin of the Ross Ice Shelf, the ASE sector, and in the Antarctic Peninsula.

**Figure 4:**
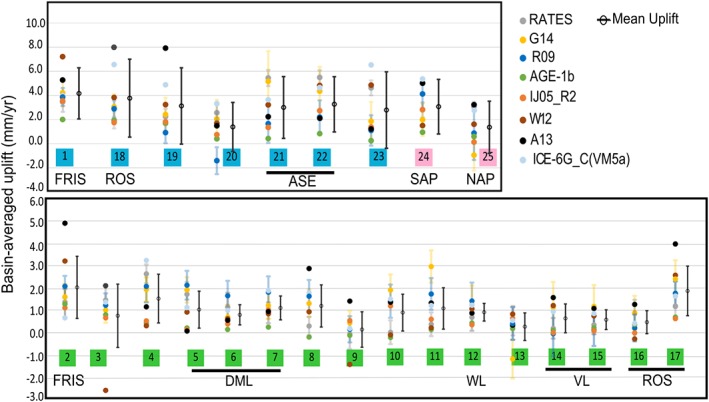
Basin‐averaged vertical displacement rates (mm yr^−1^) as computed from the different solutions. Uncertainties from inverse models are given. The global mean and standard deviation computed from the different solutions is also shown for each basin. Shadings in basin numbers indicate if they correspond to East Antarctica (green), West Antarctica (blue), or the Antarctic Peninsula (pink). Some regional indicators are provided: FRIS, Filchner‐Ronne Ice Shelf; DML, Dronning Maud Land; WL, Wilkes Land; VL, Victoria Land; ROS, Ross Ice Shelf; ASE, Amundsen Sea Embayment; NAP, North Antarctic Peninsula; and SAP, South Antarctic Peninsula.

Basin‐average uplift rates are presented for each model in Figure 4 (basins are defined following *Sasgen et al.* [2013], as shown in Figure 3).

#### 3.1.1 East Antarctica

The uplift rates predicted over East Antarctic basins are smaller than in West Antarctica and have a small variability between solutions (normally most models lying within 1*σ* interval from the mean). This is due to lack of detail in ice history going into forward models, higher rock density values in this region used in inverse models, and, for RATES, a large correlation length considered as a prior for GIA [*Zammit‐Mangion et al.*, 2015a; *Martín‐Español et al.*, 2016]. Some exceptions are found: (i) basin 2, where the model A13 predicts uplift rates of 3 mm yr^−1^ above the average, and (ii) basin 3, where the model W12 predicts a strong subsidence rate (−2.6 mm yr^−1^). The reason for (i) could be related to the deglaciation model ICE‐5G, used in A13, which likely overestimates the total ESL contribution since the LGM as being 17.5 m [*Mackintosh et al.*, 2014]. In (ii), an excessive Holocene accumulation is likely to be triggering the strong subsidence response of basin 3. Small differences in the estimated uplift rates of basins in East Antarctica produce important differences on the integrated apparent mass change due to its large area. For instance, the uplift modeled by A13 over basins 2 and 3 (comprising a total area of 2.3 × 10^6^ km^2^) leads to a total induced‐mass change of approximately 10 Gt yr^−1^ above the average.

#### 3.1.2 West Antarctica

This sector presents the largest uplift rates on Antarctica. In forward models, this could be due to the combination of two factors: the largest ice losses since the LGM and a faster response of the solid Earth. It is unclear why this also occurs in inverse models. In particular, most solutions predict their uplift maxima over the area surrounding the FRIS (basin 1), which also shows the largest mean uplift (4.7 ± 1.5 mm yr^−1^). Estimates from the models W12 and AGE‐1b provide the highest and the smallest basin‐average uplift rates (7.2 and 2.1 mm yr^−1^, respectively). Large uplift rates are also estimated over basin 18, located on the eastern margin of Ross Ice Shelf, with an average rate of 3.8 ± 2.2 mm yr^−1^. It is worth noting the high variability between the different estimates with those based on ICE‐5G or ICE‐6G ice history models (A13 and ICE‐6G_C (VM5a)) representing the clear upper bounds of the estimated mean uplifts (with 8.0 and 6.6 mm yr^−1^, respectively). If we exclude these two estimates, the mean and standard deviation of the remaining solutions decreases substantially (2.7 ± 0.7 mm yr^−1^).

GIA estimates over West Antarctica present a large variability between different models (Figure 4). Over the ASE, the predicted mean uplift rates vary from 4.9 ± 2.0 and 5.5 ± 1.1 mm yr^−1^ in models such as G14 or RATES to 0.8 mm yr^−1^ as estimated by AGE‐1b (these are average values for the region comprising basins 21 and 22). We note that the model AGE‐1b estimates relatively low uplifts in most of the basins of West Antarctica. However, given the relatively small size of the basins in this region, the variability in those uplift estimates does not have a strong impact on the correspondent apparent mass change; therefore, the impact on the regional mass balance is small. For example, there is a difference of 4 Gt yr^−1^ apparent mass change between G14 and AGE‐1b estimates in a region with an overall annual mass loss of 83 ± 5 Gt yr^−1^ over 1992–2013 [*Sutterley et al.*, 2014].

#### 3.1.3 Antarctic Peninsula

The estimated uplift rates in the Antarctic Peninsula also show large variability between different solutions. The average uplift rate estimated over SAP (3.2 ± 1.6 mm yr^−1^) is a factor of 2 larger than the average rate over the NAP (1.6 ± 1.5 mm yr^−1^). Outputs from global models (A13 and ICE‐6G_C (VM5a)) provide the strongest uplift rates in both the SAP and the NAP. The estimate from RATES also predicts a strong uplift at the NAP (3.2 ± 0.5 mm yr^−1^) , in close agreement with global models, although not one as strong as that modeled in the detailed regional study of *Nield et al.* [2014]. The lowest uplift rate is provided by AGE‐1b in the SAP. At the NAP, subsidence rates are uniquely estimated by G14. Note that inverse methods are likely to present problems in regions such as the NAP, due to the low resolution of GRACE and the large uncertainties of altimetry measurements in regions of high relief. As opposed to RATES, G14 does not include GPS rates to constrain their solution. In addition, the “LPZ bias” correction applied in G14 (unlike R09) will contribute to the subsidence.

### 3.2 Assessing and Comparing Existing GIA Models With GPS Vertical Velocities

In order to assess the performance of the GIA models, we compare the estimates with a set of elastic‐corrected vertical velocities measured with an independent set of 67 GPS stations over the period 2009–2014 (Figure 1c). We corrected the GPS‐derived vertical velocities for the elastic deformation using the outputs of the elastic model (see section 2.2) for the years 2009–2013. All GPS uplift rates referred to herein are elastic corrected. Since mass balance information was not available for 2014, we assumed the same rate as 2009–2013. It has been recently shown that parts of Antarctica are experiencing ongoing postseismic deformation [*King and Santamaría‐Gómez*, 2016]. Here we assume that the vertical expression of any associated subsidence is limited to the region near to Dumont d'Urville (Figure 1).

It should be noted that the models ICE‐6G_C (VM5a) and RATES constrain their solutions with GPS velocities, so it should be reasonable to expect these approaches to fit the validation GPS data better than the others. Unlike in other studies [e.g., *Gunter et al.*, 2014], no mean network bias has been removed before computing these statistics; therefore, there could still be a bias between GPS uplift rates and modeled rates because the GPS reference frame could differ from the center of mass of the Earth. Errors in the origin rate of International Terrestrial Reference Frame 2008 will map into errors in vertical motion at the level of up to ∼0.5 mm yr^−1^ at high latitudes [*Argus*, 2012].

The weighted mean (WM), weight root‐mean‐square (WRMS), and the median value of the residuals summarize the degree of agreement between the observed uplift rates and the modeled GIA outputs. The WM and WRMS are calculated as

(1) $$\text{WM}=\frac{\overset{67}{\underset{i=1}{\sum }}(p_{i}-o_{i})w_{i}}{\overset{67}{\underset{i=1}{\sum }}w_{i}},$$

(2) $$\text{WRMS}=\sqrt{\frac{\overset{67}{\underset{i=1}{\sum }}{\left(p_{i}-o_{i}\right)}^{2}w_{i}}{\overset{67}{\underset{i=1}{\sum }}w_{i}}},$$

where {*p*_*i*_} and {*o*_*i*_} are the predicted and observed uplift rates, and {*w*_*i*_} is the weighting factor based on the GPS measurement errors at each site

(3) $$w_{i}=\frac{1}{c_{i}{\left(\sigma_{i}^{\text{GPS}}\right)}^{2}},i=1,\ldots ,67,$$

where { $\sigma_{i}^{\text{GPS}}$} is the measurement error at each GPS site and

(4) $$c_{i}=\overset{67}{\underset{j=1}{\sum }}\exp\left(\frac{-d_{\mathrm{ij}}}{l}\right).$$

In (4), *d*_*ij*_ is the *i*th, *j*th element of the distance matrix *x* corresponding to the 67 GPS locations, while *l* is a scale parameter used to deweight stations that are in proximity of other stations. Some regions, such as the NAP and McMurdo, concentrate a large number of GPS sites, yet all spatial estimates of GIA, and hence the (unobserved) error, are spatially coherent. Failure to account for this correlation would bias WM in (1) and WRMS in (2) toward those in which several GPS stations are present. We assume a value for *l*= 250 km, obtained by fitting an exponential semivariogram to the uplift rates measured at the GPS stations and estimating the range parameter.

**Figure 5:**
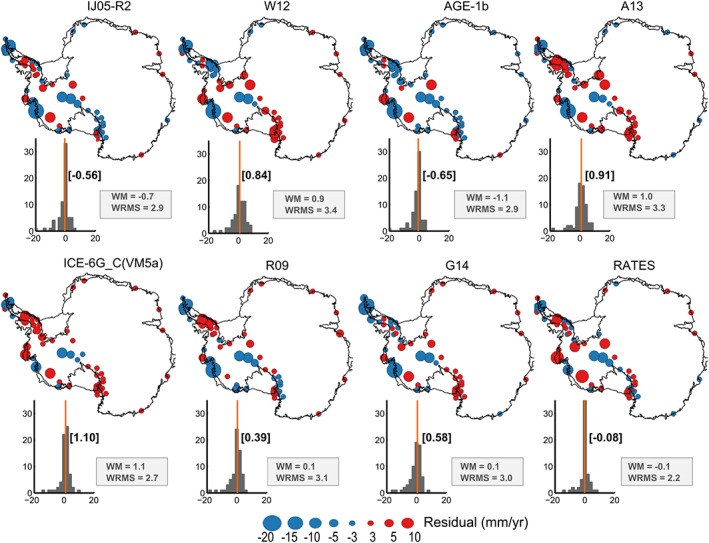
Discrepancies between the modeled and the observed GIA uplift rates estimated from different solutions in mm yr^−1^ computed at each GPS site. Red circles indicate places where the estimated GIA rates overestimate the observed velocities from GPS; blue circles indicate the converse. Histograms show the summary statistics WM and WRMS defined in (1) and (2) and are based on the weights in (3), which account for spatial correlation in the error estimation. Median values are indicated in brackets.

Figure 5 shows the differences between the estimated and observed rates of bedrock uplift at each GPS location. By examining the spatial patterns of these discrepancies, we find some common features to all solutions. For instance, the region with the largest misfits across all solutions is the ASE sector. Furthermore, there is a systematic underestimation of the uplift rates over the GPS sites *BACK* (2) and *BERP* (4) in this region (discussed in section 4.1.3). Observations at the GPS sites located near the eastern end of the Transantarctic Mountains (*FALL* (19), *BENN* (3), *WHTM* (65) are also systematically underestimated by the GIA solutions, although reasons for this bias are unclear. For the complete set of GPS observations, the weighted mean residuals tend to be smaller in those GIA solutions obtained from inverse methods than that obtained from forward models. The misfit distributions shown in the histograms do not suggest strong biases in any of the analyzed solutions for the complete set of observations. We find, however, biases on a subregional scale (see Table 2). The global solutions ICE‐6G_C (VM5a) and A13 have the largest median and WM values. The RATES GIA estimate has the smallest WRMS from all the considered approaches.

**Table 2:** The WM and WRMS of the Residuals, in mm yr^−1^, Between the Modeled and (Elastic Free) Observed Uplift Rates on GPS Sites Over Different Regions: North Antarctic Peninsula (NAP), South Antarctic Peninsula (SAP), Amundsen Sea Embayment (ASE), Margins of the Ross and Filscher‐Ronne (FRIS) Ice Shelves, and Along the Margins of East Antarctica (EA)^a^

	NAP (11)		SAP (9)		ASE (4)		Ross (25)		FRIS (5)		EA (7)	
Model	WM	WRMS	WM	WRMS	WM	WRMS	WM	WRMS	WM	WRMS	WM	WRMS
IJ05R2	−4.4	5.7	0.3	1.5	−3.5	9.6	−0.5	1.2	0.4	2.0	0.2	0.8
W12	−2.8	4.2	−2.1	2.8	−0.1	8.8	2.0	2.8	1.7	4.0	0.7	0.8
AGE1b	−4.0	5.2	−1.6	2.3	−4.3	9.8	−0.4	1.1	−1.3	2.3	−1.1	1.4
ICE6G	−1.7	3.8	3.1	3.2	1.6	9.1	1.3	1.6	2.3	2.4	−0.3	0.9
A13	−1.6	4.4	2.5	4.0	−3.3	9.3	1.4	1.9	1.7	3.5	0.4	0.6
R09	−4.1	5.9	2.1	2.6	−3.3	9.3	0.1	1.2	0.7	1.4	0.8	1.1
G14	−5.4	6.4	−0.1	0.8	−2.2	7.8	1.2	1.6	0.9	2.0	0.4	1.5
RATES	−1.3	3.2	0.2	1.3	0.1	7.7	−0.1	1.0	0.3	1.9	0.1	0.3

a The number of stations falling within each region is stated in parenthesis. The sum of the stations lying within specific regions does not match the number of total stations as some stations where not allocated to a specific region.

Table 2 shows the WM and WRMS error, respectively, on a subregional scale. GPS stations lying within each subregion are shown in Figure 1c. We find a negative bias over the NAP, which is consistent across solutions, meaning that all approaches underestimate the uplift rates in this region. Uplift rates at the ASE sector are also underestimated by most of the solutions. Exceptions are found in the RATES and ICE‐6G_C (VM5a) models, although this misfit is dominated by stations peripheral to the region (*LPLY* (36) and *THUR* (60)). Maximum uplift rates predicted in most forward and inverse models are located over the Filchner‐Ronne and Ross ice shelves in (Figure 2). However, those solutions predicting the highest uplift rates in these regions (W12, A13, G14, and ICE‐6G) overestimate the measured rates at the GPS sites.

## 4 Discussion

### 4.1 Regional Analysis and Interpretation of the GIA Uplift Rates

#### 4.1.1 Weddell Sea

The area surrounding the FRIS, in the Weddell Sea Embayment, is characterized by intermediate values of crustal thickness in the transition between East and West Antarctica and moderate mantle viscosity values [*An et al.*, 2015; *Heeszel et al.*, 2016]. The region is constrained by 3 and 1 GPS sites on its western and eastern margins, respectively. The average uplift rate of all four sites (Figure 1c) is 3.4 ± 1.8 mm yr^−1^. Most of the available GIA solutions overestimate the observed vertical rates, where the forward models W12, ICE‐6G_C (VM5a), and A13 present the strongest positive biases relative to the GPS uplift rates. Ice load history adopted by forward models assume a monotonic retreat pattern in this area. However, a revised Holocene ice‐loading history for this region has been presented [*Bradley et al.*, 2015] featuring a late Holocene readvance (and hence loading increase), resulting in a reduction in the present‐day GIA uplift rates.

A gradual gradient decreasing in the W‐E direction over this ice shelf has been observed by GPS, and it is captured in some GIA solutions, such as RATES, G14, R09, and IJ05_R2, in agreement with the viscosity gradient between East and West Antarctica shown in models [*van der Wal et al.*, 2015]. Other forward models, W12a and ICE‐6G_C (VM5a), predict their largest uplift rates over the central inner part of the ice shelf. This could be a result of errors in ice history or the simple linear 1‐D Earth structure that are adopted [*van der Wal et al.*, 2015].

#### 4.1.2 Ross Sea

This region is characterized by a strong lateral gradient that correlates with the sharp spatial gradient of modeled mantle viscosities based on seismic shear‐wave measurements [*An et al.*, 2015; *Heeszel et al.*, 2016]. The highest GPS uplift rates take place over the western margin of the ice shelf, where lower viscosities are inferred, although the lack of outcrops on the eastern side prevent GPS measurements of bedrock uplift there. The model W12 obtains the largest WRMS residual and positive WM in this region (Table 2), suggesting that the model contained too much ice at the LGM or the viscosity value used for the upper mantle is too high for this region (values of upper mantle viscosity used in W12 are higher than in IJ05_R2 and ICE‐6G_C(VM5a)). The potential for late Holocene loading changes to affect the observed rebound in this region is discussed by *Nield et al.* [2016]. A13, even resolving for 3‐D viscosity profiles, still produces a significant overestimate. The reason could rely on the fact that it is based on ICE‐5G, which features large post‐LGM ice loss in this region, or the large uncertainties in modeling 3‐D rheology [cf. *van der Wal et al.*, 2015].

#### 4.1.3 Amundsen Sea Embayment Sector

The ASE sector has been observed to rise at very high rates at three GPS sites: *BACK*, *BERP*, and *TOMO* at 9.1 ± 1.5, 19.7 ± 1.3, and 18.1 ± 0.4 mm yr^−1^, respectively (observations at the TOMO site were excluded from the study due to some anomalous motion of this site). Further evidence was provided by *Groh et al.* [2012] based on two different campaign measurements at three sites. Except for ICE‐6G_C (VM5a) and RATES, none of the examined GIA outputs reflect the uplift in this area to the extent that is observed at the GPS sites, producing a negative bias in the estimates (Table 2). Seismic evidence suggests a very low viscosity in the upper mantle of this region, on the order of  10^18^ Pa s [*An et al.*, 2015; *Heeszel et al.*, 2016], which would produce a very rapid response to ice loss with smaller spatial scale. Forward models consider typical upper mantle viscosities that are 2 orders of magnitude larger than this and do not consider loading changes in the last several thousand years, which could explain why they underpredict the uplifts of this area. Inverse models rely on assumptions of rock density values to estimate the GIA uplift. If these are too high, it might lead to underestimated uplifts in the region. We explore this further in section 4.5. Overall, the GIA models W12 and RATES produce the least biased results in the ASE, although all models show large WRMS, and hence, this low bias does not suggest overall accuracy but more likely cancelation of errors in computing the mean. It should be noted that the RATES estimate includes the GPS observations used in *Groh et al.* [2012] to constrain the solution, although these GPS sites are not included to compute the validation statistics presented in this analysis.

#### 4.1.4 Antarctic Peninsula

There are contrasting results for the NAP and the SAP. When comparing to GPS observations, we find that vertical rates predicted over the NAP are underestimated by all the GIA solutions analyzed in this study. Large biases are found in inverse solutions G14 and R09, which probably reflects the scarcity of altimeter data coverage in this region and the complexity of obtaining reliable data over areas of high relief. Despite the difficulties inherent to the region, the inverse solution from RATES performs quite well in the NAP, benefiting from the incorporation of GPS constraints in the solution. Some models (RATES, ICE‐6G_C (VM5a), and A13) estimate notably large uplifts over the northernmost part of the peninsula. However, *Nield et al.* [2014] shows that the NAP uplift rates can be almost entirely explained by low‐viscosity mantle and mass loss since the breakup of Larsen B Ice Shelf. Their modeling produced uplift rates that are larger and more spatially concentrated than those predicted by the models here and have a quite different origin. The pattern of observed GPS rates reflects largest uplifts over the NAP than over the SAP. This is in agreement with *Wolstencroft et al.* [2015] that suggested an effective upper mantle viscosity that is moderately low in the SAP region, although not as low as the NAP. Only the GIA outputs from W12 and RATES model this pattern of larger vertical uplifts over the NAP than over the SAP.

### 4.2 GIA Mass Change Correction

**Figure 6:**
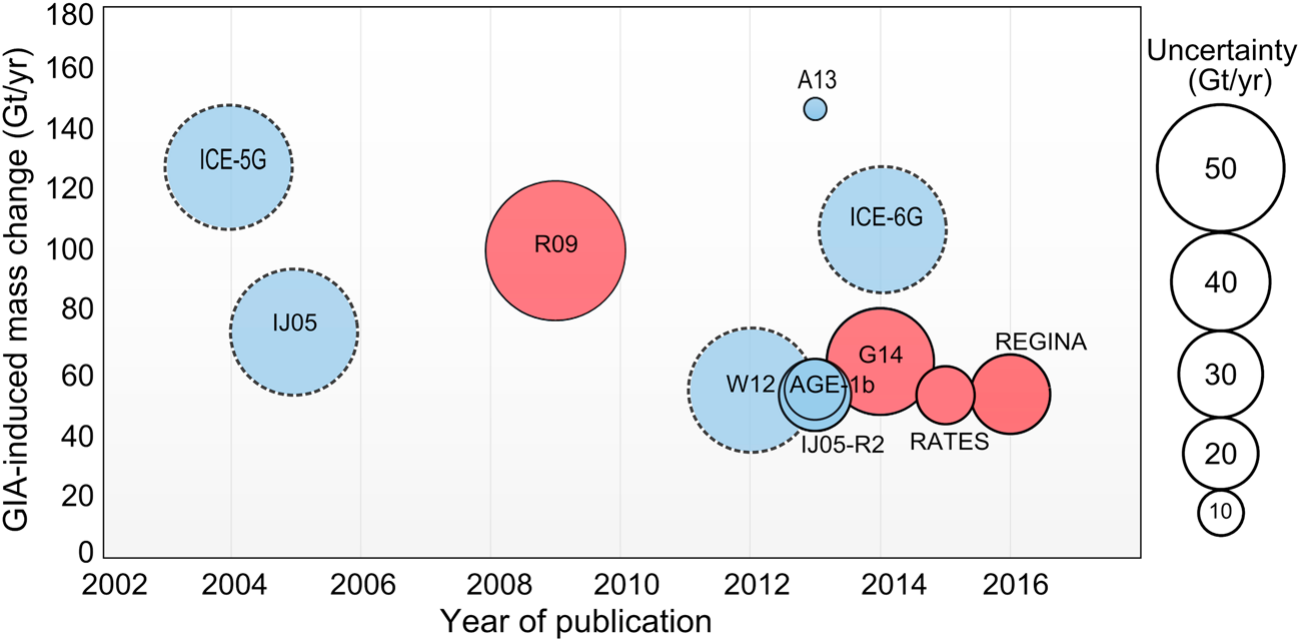
Temporal evolution of mass correction estimates from GIA models. Forward and inverse models are shown in blue and red, respectively. The radius of the circle indicates the uncertainty or range of estimates given for each model. Models where uncertainties are not available are shown with dashed circles.

Figure 6 summarizes the total GIA‐induced mass change correction to GRACE data for Antarctica, as computed by different authors. We also include here a further inverse solution from the Regional glacial isostatic adjustment and CryoSat elevation rate corrections in Antarctica (REGINA) project (unpublished, data available at http://www.regina-science.eu/). The REGINA estimate is obtained by performing a joint inversion of multiple space‐geodetic data and in situ GPS stations, using viscoelastic response functions to simulate the Earth structure in Antarctica.

The total GIA‐induced mass change seems to be following a downward trend with the most recent studies. An exception is the model A13 [*Geruo et al.*, 2013] which is based on ICE‐5G. Generally, global GIA solutions (such as ICE‐6G_C (VM5a) and A13) provide larger GIA contributions to the apparent surface mass change. On the other hand, forward models which are forced by regional ice history models (IJ05_R2 and W12a) produce smaller GIA‐induced mass changes. Overall, the GIA‐induced mass changes from the inverse solutions G14 and REGINA and RATES compare well with the existing forward models IJ05_R2, W12 and the hybrid approach AGE‐1b. The total GIA‐apparent mass change derived from different models has a standard deviation of 30 Gt yr^−1^ (excluding the estimate from ICE‐5G (VM2) which has been updated in ICE‐6G_C (VM5a)). As shown in Figure 4, the region with the largest variability in the modeled uplift rates from the different GIA solutions is the ASE region. This is also the region where all solutions consistently fit the GPS uplift rates badly. However, in spite of the strong disagreement, the overall impact on GRACE‐derived mass balance of West Antarctica is small (standard deviation of ±2.2 Gt yr^−1^). We discuss that differences in the uplift estimates over large basins of East Antarctica become more critical for the mass balance of the ice sheet in section 4.4.

### 4.3 Holocene Accumulation Over East Antarctica

Over the large region of the interior of East Antarctica, there are no GPS data available to help constraining the GIA estimates due to very limited occupation of the few remote outcrops away from the coastal margin. Due to its large size (around 10 × 10^6^ km^2^), even a small misinterpretation of the GIA signal will cause a strong deviation of the actual mass balance of the ice sheet (1 mm yr^−1^ of GIA over East Antarctica is equivalent to approximately 45 Gt yr^−1^).

A recent study using altimetry data reported a mass balance of 82 ± 25 Gt yr^−1^ for the Antarctic ice sheet over 2003–2008 [*Zwally et al.*, 2015]. This estimate is much larger than any other based on either gravimetry, altimetry, or geodetic techniques. [*Scambos and Schuman*, 2016]. To reconcile their estimate with GRACE measurements, *Zwally et al.* [2015] proposed that current GIA models could be adjusted by removing a mean rate of −1.6 mm yr^−1^ in East Antarctica. This hypothesis is supported by the assumption of a large dynamic thickening occurring over the interior East Antarctica as a response to a persistent accumulation trend during the Holocene. The current average GIA over East Antarctica is 0.9 mm yr^−1^ (considering all the models assessed in this study), which is equivalent to 40 Gt yr^−1^ using a rock density of 4500 kg m^−3^. The average GIA rate proposed by *Zwally et al.* [2015] would be −0.7 mm yr^−1^ which is equivalent to a negative apparent mass change of −32 Gt yr^−1^, far from any result derived from the GIA models available. Furthermore, it should be noted that models such as W12 and IJ05_R2 already account for Holocene accumulation increases.

### 4.4 Variants to RATES Solution

#### 4.4.1 Decreasing the Length Scale Over West Antarctica

**Figure 7:**
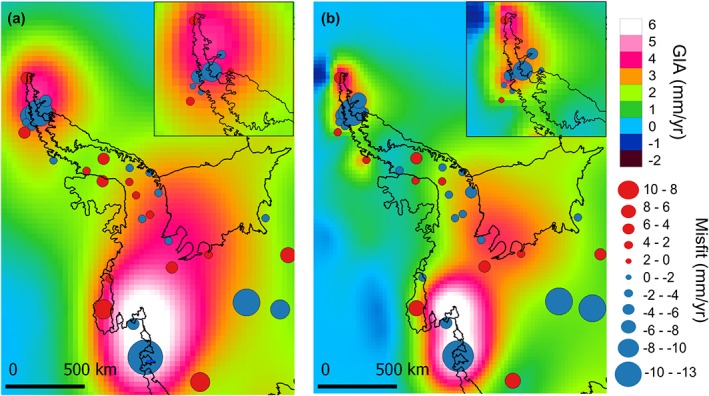
Estimated GIA uplift rates from RATES and GPS misfits (a) using the original spatial wavelength as in *Martín‐Español et al.* [2016] and (b) decreasing the spatial wavelength attributed to GIA over West Antarctica to 250 km.

The largest WRMS produced in the RATES solution take place over the ASE sector and the NAP (Table 2). These are areas of thin crustal thickness and low viscosity where recent ice loading changes are likely to be reflected in the GIA signal, reducing the characteristic length scale of those regions (i.e., 100 km in the NAP [*Nield et al.*, 2014]). Therefore, large misfits of GIA in these regions could indicate that the length‐scales assumed in *Martín‐Español et al.* [2016] (derived from a correlation analysis of the strong rheology version of IJR5‐R2) over these places exceed the wavelengths of the processes taking place. On this basis, we use a new correlation structure for GIA which employs a decrease in the spatial length scale over West Antarctica. We consider a spatial wavelength of 250 km at the northern‐most tip of the NAP (500 km was previously assumed), which linearly increase up to 1700 km over East Antarctica. This modification leads to a spatial concentration of uplift, with larger maxima (Figure 7) but an overall reduction of the mean uplift rates. The overall WRMS residual from the GPS comparison modestly decreases from 2.2 to 2.1 mm yr^−1^. On a subregional scale, improvements are produced over the ASE sector (7.7 to 6.9 mm yr^−1^), the Weddell Sea region (1.9 to 1.7 mm yr^−1^), and the SAP (1.3 to 1.1 mm yr^−1^). Due to the overall decrease of the uplift rates, there is also a reduction of 10 Gt yr^−1^ in the total GIA‐induced apparent mass change, from 55 ± 8 Gt yr^−1^ to 45 ± 7 Gt yr^−1^.

#### 4.4.2 Sensitivity of the RATES GIA Model to the GRACE Solution

**Figure A1:**
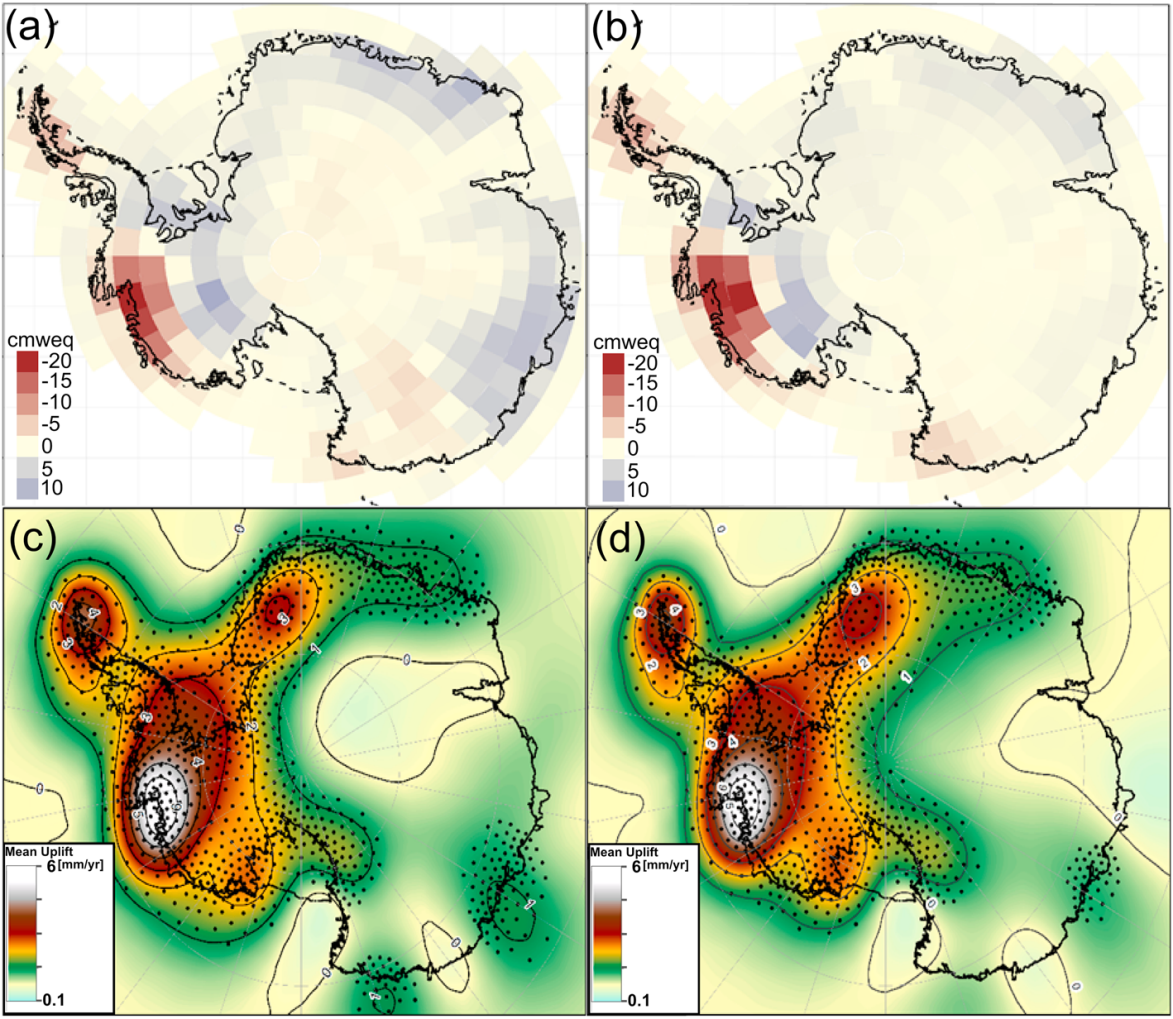
Mass anomalies for Antarctica in cm water equivalent over 2003–2009 (period for which GIA has been estimated) using two different GRACE solutions (a) RL02v15 and (b) *Andrews et al.* [2015] and the (c and d) derived GIA estimates using a Bayesian statistical approach are shown, respectively.

To assess the influence the choice of GRACE solution has on the predicted GIA uplift rates from the RATES solution, we compare results obtained from two mascon monthly solutions derived independently from different centers and software *Andrews et al.* [2015] (hereafter denoted A2015) and the RL02v15 version of *Luthcke et al.* [2013]. Further details and discussion of differences can be found in the Appendix. The resulting rates of vertical displacement due to GIA obtained from the gravimetry solutions agree well (see Figure A1), showing robustness of the method to different GRACE analysis approaches. East Antarctica is more sensitive to small changes in the GRACE field. This is due to the low rates of accumulation that this region is subject to, with a signal noise ratio close to unity. The major discrepancy occurs over Mac Robertson Land (basins 9 and 10), where the RL02v15 solution detects a larger positive anomaly than *Andrews et al.* [2015], driving subsidence rates over a larger portion of East Antarctica.

### 4.5 Limitations and Uncertainties

The comparisons presented in this work are valid upon the assumption of an error‐free elastic field being removed from the GPS velocities. However, variations from the adopted elastic Earth model could produce vertical differences of 10% in the presence of localized changes [*Dill et al.*, 2015]. Another source of error is the uncertainty in the mass balance which feeds the elastic model. The elastic correction applied has been estimated using the posterior annual mass trends derived from a Bayesian framework [*Martín‐Español et al.*, 2016]. We checked the influence of the posterior uncertainty on the elastic correction and how it propagates to the results above. This was done by simulating an ensemble of 100 mass balance realizations from the posterior distribution and running the elastic model with each of these loading distributions. The variances of the simulated elastic responses at the GPS locations were then added to the measurement error { $\sigma_{i}^{\text{GPS}}$} in equation (3). The resulting WM and WRMS residuals are given in Table 3 where we see that the residuals are largely insensitive to the uncertainties in the mass balance. We note that this test does not fully accommodate for the variability in elastic deformation as the RATES solution cannot resolve mass changes that may occur over very small spatial scales and hence acts to dampen larger values.

**Table 3:** Sensitivity of the WM and WRMS Residuals in mm yr^−1^, Between the Modeled and Observed GPS Uplift Rates^a^

	Results		Elastic‐CS2		Elastic‐MB Error	
	WM	WRMS	WM	WRMS	WM	WRMS
IJ05R2	−0.7	2.9	−0.4	3.2	−0.6	2.8
W12	0.9	3.4	1.1	3.7	1.0	3.4
AGE1b	−1.1	2.9	−0.9	3.0	−1.1	2.8
ICE6G	1.1	2.7	1.3	3.1	1.1	2.7
A13	1.0	3.3	1.2	3.6	1.0	3.2
R09	0.1	3.1	0.3	3.5	0.1	3.0
G14	0.1	3.0	0.3	3.4	0.1	2.9
RATES	−0.1	2.2	0.2	2.4	−0.1	2.2

a We show the original results presented earlier, the results where the elastic effects have been estimated from a CS2‐mass balance estimate (Elastic‐CS2), and the results when accounting for mass balance uncertainty in the elastic model (Elastic‐MB Error).

Mass rates derived from the Bayesian framework are robust and have good spatial and temporal resolution for the overlapping period to the GPS uplift rates. Some other studies have used average mass trends values by uniformly distributing mass losses over specific basins or adopting different flux epochs that do not match the period of observed velocities [e.g., *Thomas et al.*, 2011, supporting information] to compute the elastic deformation. However, we find that differences between distinctive mass flux epochs are not negligible (Figure 1), and it is therefore essential to apply a dynamic elastic correction [see also *Nield et al.*, 2014; *Wolstencroft et al.*, 2015].

We note a dependency between the elastic correction applied to the GPS uplift rates and the RATES solution which is coestimated along with the mass balance estimates. However, we note that the time period for which the GPS velocities are used (2009–2014) does not overlap with the time frame for which RATES GIA solution is derived (2003–2009). We consider the importance of this partial dependency by examining the sensitivity of the results presented in this study to an independent elastic correction derived from CryoSat‐2. Elevation changes are obtained from Cryosat‐2 measurements for the period 2010–2013. These are corrected for firn densification processes using the model by *Ligtenberg et al.* [2011]. The density assumption required for volume to mass conversion is achieved by determining which process is the main driver of the elevation change. Dynamic losses occurring at the density of ice are associated to high‐velocity regions for which we apply a velocity threshold of 100 m yr^−1^. An exception is made in the Kamb Ice Stream, where dynamic elevation changes are known to occur in spite of low velocities [*Retzlaff and Bentley*, 1993]. In this area we allow for dynamic changes to occur when d*h*/d*t* >30 cm yr^−1^. Outside dynamically active regions, we assume elevation changes to occur at the density of snow which is obtained from RACMO2.3. Mass loads are estimated over a 10 km resolution grid to reconstruct the elastic correction. The results from the assessment of the GIA solutions with the GPS velocities corrected with the CryoSat‐2‐derived elastic correction are found in Table 3. They show a small increase in the WRMS which is consistent in all solutions. However, we do not observe any favoritism to the RATES GIA solution in this analysis.

Finally, we explore the sensitivity of the RATES GIA solution to the value used to represent the solid Earth density. Considering that RATES underestimates the GIA uplift rates in some regions of West Antarctica, we recompute the GIA solution using a lower value for the rock density, 3400 kg m^3^, corresponding to a shallow depth of 200 km according to the inferred densities from the preliminary reference Earth model [*Dziewonski and Anderson*, 1981]. We find improvements in the WRMS residual from the GPS comparison. Over the SAP, we found an 11% decrease of the WRMS, 8% decrease over FRIS, 5% over ASE, and 2% over NAP. No significant changes were obtained over the Ross Ice Shelf.

## 5 Conclusions

In this study we have assessed the eight most up to date GIA models for Antarctica using a GPS network of elastic‐corrected vertical velocities. The analysis has revealed specific regional biases present in the GIA estimates. Uplift rates over the ASE sector and over the NAP are underestimated by all available GIA solutions. Thinner lithosphere and lower viscosity Earth structure correlates with more rapid and shorter length‐scale uplifts, and we suggest that these regions both are underlain by low viscosity mantle, in agreement with the findings of *Nield et al.*[2014] for the NAP and the suggestion of *Groh et al.* [2012] for the ASE. These regions of low viscosity are also significantly affected by present‐day changes in ice loading which is not accounted for in millenial scale forward models that do not consider loading changes in the recent several thousand years. On the other hand, limitations on the resolution and scarcity of data coverage over areas with pronounced relief leads empirical methods based on altimetry and gravimetry to likely underpredict the uplift rates. We find an improvement from the GPS comparison when decreasing the value of the solid Earth density over West Antarctica in the RATES GIA solution, as would be expected if GIA in these regions was dominated by mass redistribution in the relatively shallow mantle. The GPS comparison reveals that the uplift rates over the Filchner‐Ronne and Ross ice shelves (where maximum uplifts are predicted in most GIA models) are overestimated by those models predicting the largest uplifts in these regions. Forward GIA models have ice histories dominated by ice loss following the LGM and often rely on 1‐D Earth models with a linear Maxwell rheology, and it is becoming more evident that a 3‐D Earth structure with more complex rheology (e.g., power law) will be necessary to correctly capture the viscoelastic solid Earth response to changes in surface [*van der Wal et al.*, 2015].

Estimates of total GIA‐induced mass change affecting gravimetry methods derived from both inverse and forward methods tend to converge toward 60 Gt yr ^−1^ in the newer solutions. However, there is a large variability of the GIA estimates on a regional scale. The interior of Antarctica, for which there are large uncertainties, plays a key role in terms of providing an accurate correction for gravimetry methods, despite its small magnitude of vertical displacement. Therefore, estimating an accurate present‐day GIA uplift rate still remains an open field of research, and more measurements are needed to constrain and improve the solutions of both the timing of the deglaciation history and the measure of current uplift.

## Supporting information



Supporting Information S1

Table S1

## Appendix A Processing of GRACE Mascon Solutions

### A.1

We used two independent mascon monthly solutions A2015 [*Andrews et al.*, 2015] and the RL02v15 version from *Luthcke et al.* [2013]. The two mascon solutions are conceptually similar being based on short‐arc GRACE solutions from K‐band range‐rate data (KBRR) only, geographic spatial constraints, baseline state parameterization [*Rowlands et al.*, 2002], and accelerometer calibration from 24 h arcs. However, the a priori force models differ in the gravity field (GIF48 v GGMOC2), ocean tide models (FES2004 v GOT4.7), and the use of forward modeling for GIA and land hydrology in RL02v15. A2015 utilized 60 min short arcs for the GRACE tandem pair (cf. daily arcs in RL02v15) solving for three orbital baseline parameters and the mascon parameters with normal matrices combined to form daily and then monthly solutions. It is noted that RL02v15 used 1° arcdeg equal area sampling which is half the 2° of A2015. The main advantage of the higher spatial resolution is more accurate delineation of Antarctic basins. In addition, in RL02v15 an iterative procedure was applied which the authors show leads to significant improvement in the mascon solutions in their approach. Both methods incorporate a damping parameter for the constraint matrix. Varying this parameter has impact on the solution noise. In RL02v15 a constant damping factor was applied, while A2015 used a damping factor that increased during the period of reduced thermal control of GRACE after February 2011. Poor thermal control is the result of changes in accelerometer heating, brought on by decreasing battery performance [*Kim and Tapley*, 2015]. A crucial aspect of GRACE data analysis is quality control of the KBRR, accelerometer, and attitude data. Both solutions pay particular attention to this issue, but the independence of the approaches will mean that different data quality control is inevitable. An ensemble empirical mode decomposition (EEMD) 730 filter was applied to the mascon solutions (after restoration of GIA RL02v15) to remove interannual variability.
