# Supplementary material for: An assessment of forward and inverse GIA solutions for Antarctica
Source: J Geophys Res Solid Earth. 2016 Sep 29;121(9):6947–65. doi: 10.1002/2016JB013154 (PMC5111427; doi:10.1002/2016JB013154)
Supplement: Supplementary file 1 — Supporting Information S1 [file JGRB-121-6947-s001.pdf]

AGU PUBLICATIONS

Journal of Geophysical Research: Solid Earth

Supporting Information for

An assessment of forward and inverse GIA solutions for Antarctica

Alba Martín-Español[1], Matt A. King[2], Andrew Zammit-Mangion[3], Stuart B. Andrews[4], Philip Moore[4], Jonathan L. Bamber[1]

[1]School of Geographical Sciences, University of Bristol, Bristol, BS8 1SS, UK

[2]School of Land and Food, University of Tasmania, Australia

[3]National Institute for Applied Statistics Research Australia (NIASRA), School of Mathematics and Applied Statistics, University of Wollongong, Australia

[4]School of Civil Engineering and Geosciences, Newcastle University, UK

Contents of this file

Caption for Table S1

Introduction

This supporting information includes Table S1 detailing the GPS stations used to carry out the assessment presented in the main text and the observed (elastic-corrected) and predicted rates from each GIA solution at each site.

Caption Table S1. GPS sites specifications including their geographical locations, observation period and Digital Object Identifiers (when available). An internal ID has been given to each GPS site and is refered to in the main text. Observed uplift rates (after applying the elastic correction*) and predicted rates (in mm yr^{-1}) from each GIA solution included in this study are given for each site.

*Annual grids of the elastic correction are available on request.
